# The Role of Rumination and Worry in the Bidirectional Relationship Between Stress and Sleep Quality in Students

**DOI:** 10.3390/ijerph22071001

**Published:** 2025-06-25

**Authors:** Ana Petak, Jelena Maričić

**Affiliations:** Faculty of Croatian Studies, University of Zagreb, Borongajska Cesta 83d, 10000 Zagreb, Croatia

**Keywords:** panel design, rumination, sleep quality, stress, students worry

## Abstract

Poor sleep is strongly associated with stress; however, the mediators of this relationship are not well understood. Cognitive arousal in the form of rumination and worry may mediate this relationship, but previous studies have primarily focused on patients with insomnia or employed cross-sectional designs. This study aimed to explore the causal relationship between sleep quality and stress using two-wave longitudinal data and examining the theoretical assumptions of insomnia models in a healthy, non-clinical student population. Research was conducted using a paper-and-pencil method. The sample included 302 undergraduate students from the University of Zagreb. Most of the participants were female (79.8%) and aged between 18 and 33 years, representing various fields of study. The research problem was examined through multiple mediation analysis. The results confirm our initial hypothesis regarding the bidirectional relationship between stress and sleep quality, which is partially mediated by rumination. The mediating effect of worry is significant only in parallel mediation; poor sleep quality leads to increased rumination (*p* < 0.01), which in turn predicts more worry (*p* < 0.01), and greater worry predicts more stress (*p* < 0.01). The effect sizes indicate that stress has a more significant impact on sleep problems (*β* = 0.345, *p* < 0.01) than sleep problems have on stress (*β* = 0.203, *p* < 0.01). These findings are important for planning preventive activities and therapeutic interventions.

## 1. Introduction

Global meta-analyses of epidemiological data [1] indicate that over 60% of mental health problems are detected by the age of 25, with stress and sleep disturbances among the most common [2,3]. The student population is particularly vulnerable to stress [3,4,5], which may arise from relationships with peers, teachers, and parents, in addition to academic pressures [6]. Stressors associated with starting college can exacerbate pre-existing mental health difficulties or trigger the initial onset of symptoms [7]. Additionally, various concerns also arise from the specific circumstances of global crises and insecurities that young people face today. In Europe, young people experience job loss, financial insecurity, and mental health problems more often, in general, compared to older age groups [8,9]. Therefore, their tendency to experience stress is not surprising, making this particular population important for researchers. Because university students face various challenges that can profoundly affect their mental health, it is essential to investigate their mental well-being. Stress is associated with a range of mental health issues among youth, including depression, anxiety, post-traumatic stress disorder, and insomnia [10], with a particular emphasis on the relationship between sleep problems and stress [11,12,13]. Among university students, perceived stress is associated with lower sleep quality [14,15], shorter sleep duration [15], and insomnia symptoms [16]. In recent years, a decline in sleep quality has been observed among young adults [2,17]; research indicates that between 47% [18] and 60% [19] of students experience poor sleep quality. The term “sleep quality” is complex and refers to both objective aspects (such as duration and latency) and subjective aspects of sleep (such as depth of sleep and the feeling of being well-rested) [20,21]. Disturbed sleep is a possible transdiagnostic process for various psychological disorders [22,23]; it is related to cognitive and neurological functions of the brain [24,25,26] as well as internalized problems such as anxiety and depression [27,28]. Disrupted sleep can lead to poor academic performance, more risk behaviors, and declines in social, physical, and mental health [29]. Therefore, it is crucial to investigate the underlying factors that contribute to poor sleep quality, including stress. The correlation between stress and sleep has been thoroughly documented [11,12,13,30,31,32,33]. However, the specific characteristics of this relationship remain less well understood.

Studies [34,35,36,37] indicate that certain cognitive mechanisms and high general arousal may affect the relationship between experienced stress and sleep difficulties. This assumption is derived from several theories. According to the Cognitive Model of Insomnia [38], individuals with insomnia are prone to excessive worry about their sleep and the consequences of disrupted sleep on their health. This negative cognitive process in the form of worry and rumination activates autonomic arousal and consequently disrupts sleep. The Hyperarousal Model of Insomnia [39] assumes that insomnia occurs due to a combination of genetic vulnerability, stressors, dysfunctional behavior patterns, learned behaviors, and cognitive activity such as worry and rumination. Somatic, cognitive, and cortical activity results in excessive general arousal [39], which may lead to anxiety, a real sleep deficit, worry, physiological arousal, and high levels of daytime distress [38]. This creates a cycle in which the connection between stress and sleep quality is potentially bidirectional; i.e., greater stress disrupts sleep, and disrupted sleep creates additional stress. Another theory of hyperarousal [40] also suggests that arousal and coping skills may mediate the sleep–stress relationship; appraisal of stressors and a perceived lack of control lead to vulnerability to sleep problems, such as insomnia. In one study, while unhelpful beliefs about sleep were shown to predict insomnia severity in all age groups, pre-sleep arousal was the main contributor to insomnia in young adults [41]. Morin’s [40] results show that both poor and good sleepers reported similar numbers of minor stressful experiences; however, individuals with insomnia rated the impact and intensity of these experiences higher than good sleepers. Additionally, they reported greater pre-sleep arousal and perceived their lives as more stressful [40,42,43].

Since various other models (e.g., [44,45,46,47]) recognize rumination and worry as cognitive processes that increase arousal and potentially mediate the relationship between stress and sleep quality, this was also assumed to be the case in this study. Rumination refers to repetitive, prolonged, and negative thinking about feelings, worries, and distressing experiences [48,49] without taking action to positively change that state [50]. It is considered to be a transdiagnostic pathological process for various mental health problems [49,50]. Similarly to rumination, worry is a sequence of negative thoughts and images that are difficult to control, accompanied by an attempt to mentally resolve problems with uncertain outcomes, which may include one or more negative consequences [51]. Worry and rumination are two processes that involve repetitive thinking regarding negative emotional experiences and shift focus away from the present (toward the past or future). However, while worry refers to future events, rumination is focused on past failures and worries [52,53]. Despite their similarities, rumination and worry are distinct concepts with clear differences. Rumination focuses on issues related to personal worth, meaning, and loss, while worry centers on anticipated potential threats. Consequently, the conscious motivation for rumination is to gain insight into a situation, whereas the motivation for worry is to predict and prepare for potential threats [54]. Since rumination and worry are distinct processes, they exert independent and separate effects on sleep and mental health [55,56]. Therefore, it is appropriate to consider them separately. It was found that the tendency for young people to worry increased by 20% from 2001 to 2019. The most dominant concern was academic success, followed by worries about intimate relationships and the opinions of others, while financial and health concerns ranked lower [57]. Considering the academic pressures faced by young people [6,57,58], along with the challenging social and cultural context of the time in which they live in [8], it is not surprising that students often ruminate and worry about various issues.

The influence of worry and rumination on sleep disturbances is recognized by the theories mentioned above, specifically the Cognitive Model of Insomnia [38] and the Hyperarousal Model of Insomnia [39]. However, these models primarily focus on the relationship between stress and sleep quality in patients with insomnia, which raises questions about the applicability of these assumptions to other populations. According to Morin et al. [40], the stress–sleep relationship is more stable in individuals with insomnia due to a different perception of stressful experiences. Additionally, previous studies have predominantly employed cross-sectional designs (e.g., [30,31]), which limit the ability to draw more complex conclusions about the relationship between sleep quality and stress. Although rumination, worry, poor sleep quality, and stress are common factors associated with various disorders [55,59], examinations of the dynamics between these constructs are rare. Therefore, this research aims to verify certain assumptions of the Cognitive Model of Insomnia [38] and the Hyperarousal Model of Insomnia [39] regarding the influence of rumination and worry on the relationship between stress and sleep within a population of students, enhancing the existing literature on the relationship between sleep quality and stress by utilizing a panel design and examining the theoretical assumptions of the insomnia models in a healthy, non-clinical student population. We hypothesize that the relationship between sleep quality and stress is bidirectional and that it is mediated by worry and rumination. Because the above theories recognize different types of arousal underlying insomnia, partial mediation is expected.

## 2. Methods

### 2.1. Subjects and Study Design

A longitudinal two-wave study was carried out during the autumn (first wave) and spring (second wave) of the academic year 2021/2022. We assessed participants at the midpoint of both the first and second semesters of the same academic year, with an interval of approximately 3 to 4 months between the two waves. In this period, exposure to academic stress should be similar across both research conditions. Prior to the data collection, approval was secured from the Ethics Committee of the Faculty of Education and Rehabilitation Sciences, University of Zagreb (24 February 2021, approval number: 602-04/21-42/07). The study was conducted during the COVID-19 pandemic, when instruction at Croatian universities was most commonly delivered in a hybrid format, combining online and in-person classes [60]. The faculties of the University of Zagreb were selected using a convenience sampling method. Participation was voluntary and anonymous, and written informed consent was obtained from all participants. Research was conducted using a paper-and-pencil method. A total of 526 students who were present in regular university classes on the day of the research were involved in the initial phase of the study, while 594 students participated in the subsequent phase. It was feasible to match codes from both phases for 302 respondents, thereby creating a dependent sample. It is posited that the observed attrition was attributable to the varied attendance of students during the two phases of the research, which consequently led to a reduced number of questionnaires available for matching. The sample included 302 undergraduate students from the University of Zagreb who provided data from both research waves. Most participants were female (79.8%) and aged between 18 and 33 years (*M* = 20.27, *SD* = 1.68), from various fields of study. Of the participants, 63.9% estimated their socioeconomic status as average, 27.8% as above average, and 8.3% as below average. Among the participants, 7.6% reported previously confirmed diagnoses of mental health conditions, mostly (4.3%) mood disorders.

### 2.2. Measures

The Pittsburgh Sleep Quality Index (PSQI; [61]) was used to assess self-reported sleep quality. The PSQI consists of 19 items that measure qualitative and quantitative aspects of sleep, with responses measured on various scales. The results can be reported as an aggregate score or as scores across seven distinct dimensions: subjective sleep quality, sleep duration, sleep latency, sleep efficiency, sleep disturbances, use of sleep medication, and daily functioning. A cut-off value of 5 was used to distinguish between good sleepers (scores under 5) and poor sleepers (scores over 5). Cronbach’s α was 0.602 in the first wave and 0.667 in the second wave.

The Depression, Anxiety, and Stress Scales (DASS-21, [62]) was used to assess the severity of stress symptoms. The DASS contains 42 items, while the DASS-21 is a shorter version. The results can be divided into three subscales: depression, anxiety, and stress. Participants reported their level of agreement on a scale from 0 (does not apply to me at all) to 3 (applies to me completely). The Cronbach’s α of stress subscale was 0.901 in the first wave and 0.880 in the second wave.

To assess rumination, the Rehearsal Scale from the Emotional Control Questionnaire (ECQ, [63]) was used. This scale measures the degree of rumination about previous upsetting emotional experiences. The subscale consists of 14 items, with a true/false answer format. The response scale was transformed on a scale from 1—not at all true for me to 5—completely true for me, because it was shown that this change does not interfere with the psychometric characteristics of the ECQ-R [52]. A higher score indicates a greater tendency toward rumination. The reliability of the scale in the first wave of this research was 0.646, and in the second wave it was 0.690.

The Penn State Worry Questionnaire (PSWQ, [64]) was used as the measure of worry. The PSWQ consists of 16 items, with responses rated on a scale from 1 (does not apply to me at all) to 5 (completely applies to me). A higher total score on the scale indicates a greater expression of pathological worry. The reliability of the questionnaire in the first wave was 0.941, and in the second wave it was 0.940.

## 3. Results

### Data Analysis

The main research problem was examined through multiple mediation analysis (Model 6) utilizing the PROCESS macro software version 4.2 developed by Hayes [65]. Two distinct multiple mediation analyses were conducted to explore the hypothesis that rumination and worry are mediators in the relationship between stress and sleep quality on a longitudinal level, and to examine the reciprocal effect between stress and sleep quality. The bootstrap confidence interval (*CI*) was set at 95% and analysis was performed with 5000 bootstrap samples. The path was considered significant at the 0.05 level, with the lower and upper boundaries of the 95% *CI* not including zero. The effect size was interpreted in accordance with Cohen’s [66] guidelines, which classify a standardized coefficient of 0.10 as a small effect, 0.30 as a medium effect, and 0.50 as a large effect. A z-score exceeding an absolute value of 3.29 was classified as an outlier [67,68], so two outliers detected through z-scores were removed from further analysis. Little’s MCAR analysis showed that data were missing completely at random (*χ*^2^ = 45.841, *df* = 54, *p* = 0.777), with 0 to 2.3% of missing data in a single variable. Missing data was treated by listwise deletion, since this is the only method of handling missing data in the PROCESS macro [69]. Consequently, final mediation analyses were conducted on a total of 287 participants, excluding outliers and including only those with complete data on all variables included in the model.

According to the results of the *t*-test for dependent samples (Table 1), no significant differences in sleep quality and stress scores were found between the two waves of the study. However, students in the first wave were more worried and ruminated more than in the second wave. Although these differences are statistically significant, they are very small, and we used only second-wave data on worry and rumination as mediators. Before performing the main analysis, we calculated the correlations among the study variables. Stress, sleep quality, rumination, and worry were positively intercorrelated as expected in both research waves (Table 1).

Firstly, a multiple mediation analysis was used to test the indirect effect of rumination and worry on the relationship between stress and sleep quality. Sleep quality in the second research wave was predicted based on stress from the first research wave. Rumination and worry from the second wave were set mediators, since it was expected that an effect would appear between the two waves of research. The same analysis was conducted with stress from the second research wave as a criterium and sleep quality from the first research wave as a predictor. In both analyses, age, gender, and a history of mental health problems (existence of a diagnosis in the field of mental health problems) were controlled for as covariates.

**Figure 1:**
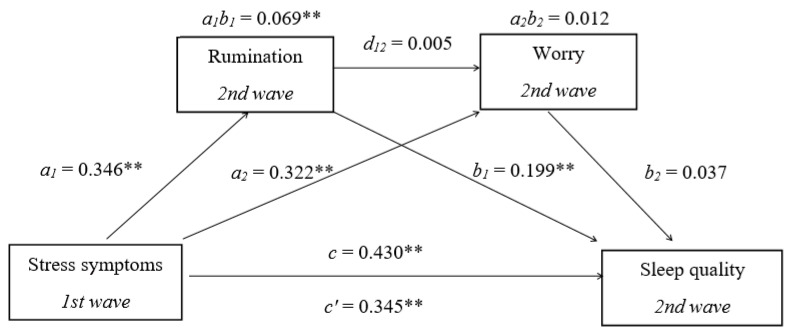
Multiple mediation model with stress symptoms (*X*), sleep quality (*y*), rumination (*M1*), and worry (*M*2). Standardized path coefficients are shown; ** *p* < 0.01, *c*—total effect coefficient.

The results of the first mediation analysis (Figure 1) indicated a significant direct path between stress and sleep, as stress symptoms were a significant predictor of sleep quality (*B* = 0.103, *SE* = 0.019, 95% *CI* [0.065, 0.141], *β* = 0.345). Stress symptoms were a significant predictor of rumination (*B* = 0.258, *SE* = 0.045, 95% *CI* [0.170, 0.346], *β* = 0.346), and rumination was a significant predictor of sleep quality (*B* = 0.079, *SE* = 0.025, 95% *CI* [0.030, 0.129], *β* = 0.199), so the indirect effect of rumination on the stress–sleep relationship was significant (*B* = 0.021, *SE* = 0.007, 95% *CI* [0.007, 0.037], *β* = 0.069). Increased feelings of stress were associated with higher levels of rumination, which in turn resulted in poorer sleep quality. Stress symptoms were a significant predictor of worry (*B* = 0.472, *SE* = 0.074, 95% *CI* [0.327, 0.616], *β* = 0.322), but worry was not a significant predictor of sleep quality (*B* = 0.008, *SE* = 0.015, 95% *CI* [−0.021, 0.036], *β* = 0.037), so the indirect effect of worry on the stress–sleep relationship was not significant (*B* = 0.004, *SE* = 0.006, 95% *CI* [−0.090, 0.016], *β* = 0.012). Rumination could predict worry (*B* = 0.739, *SE* = 0.093, 95% *CI* [0.556, 0.923], *β* = 0.376), but worry was not a significant predictor of sleep quality, so the serial mediation effect was also nonsignificant (*B* = 0.001, *SE* = 0.003, 95% *CI* [−0.004, 0.007], *β* = 0.005). Approximately 18% of the variance in sleep quality was accounted for by the predictors (*R*^2^ = 0.184). According to Cohen’s [66] guidelines, we found a medium effect size for the direct relationship between stress and sleep, as well as a small indirect effect of rumination as a mediator.

**Figure 2:**
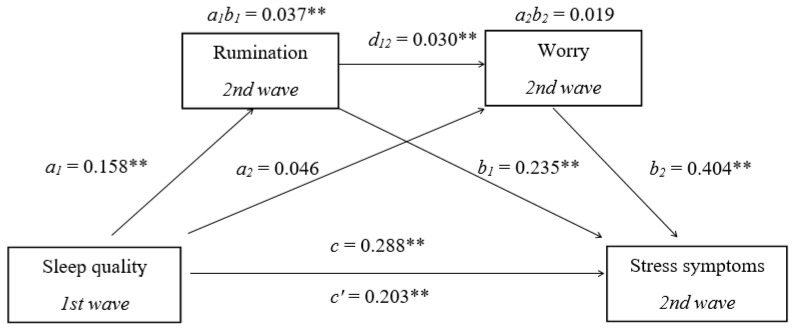
Multiple mediation model with sleep quality *(X*), stress symptoms *(y*), rumination (*M1*), and worry (*M*2). Standardized path coefficients are shown; ** *p* < 0.01, *c*—total effect coefficient.

The results of the second mediation analysis (Figure 2) indicated a significant direct path between stress and sleep; sleep quality was a significant predictor of stress symptoms (*B* = 0.687, *SE* = 0.156, 95% *CI* [0.377, 0.997], *β* = 0.203) and of rumination (*B* = 0.417, *SE* = 0.157, 95% *CI* [0.108, 0.725], *β* = 0.158), while rumination was a significant predictor of stress symptoms (*B* = 0.301, *SE* = 0.068, 95% *CI* [0.167, 0.435], *β* = 0.235), so the indirect effect of rumination on the sleep–stress relationship was significant (*B* = 0.125, *SE* = 0.056, 95% *CI* [0.025, 0.250], *β* = 0.037). Poorer sleep quality is related to higher levels of rumination, which in turn results in more stress symptoms. Worry was a significant predictor of stress (*B* = 0.264, *SE* = 0.037, 95% *CI* [0.191, 0.337], *β* = 0.404), but it could not be predicted by sleep quality (*B* = 0.239, *SE* = 0.253, 95% *CI* [−0.258, 0.737], *β* = 0.046), so the indirect effect of worry on the sleep–stress relationship was not significant (*B* = 0.063, *SE* = 0.066, 95% *CI* [−0.090, 0.016], *β* = 019). In serial mediation, rumination could predict worry (*B* = 0.919, *SE* = 0.095, 95% *CI* [0.732, 1.105], *β* = 0.467), and worry could predict stress symptoms, so the serial mediation effect was significant (*B* = 0.101, *SE* = 0.043, 95% *CI* [0.021, 0.195], *β* = 0.030). Approximately 17% of the variance in stress symptoms was accounted for by the predictors (*R*^2^ = 0.167). In this model, where sleep quality was set as a predictor of stress, the direct relationship had a small-to-medium effect size. Additionally, there was a small indirect effect of rumination as a mediator, as well as a small indirect effect of worry in the serial mediation model.

According to our results, the relationship between stress and sleep quality is reciprocal. There is a significant bidirectional path between sleep quality and stress symptoms, partially mediated by rumination. Worry was not a significant mediator of the sleep–stress relationship. In serial mediation, part of the effect of sleep quality on stress symptoms is transmitted through a mediation chain between rumination and worry—sleep quality predicts rumination, rumination predicts worry, and worry predicts stress symptoms. The magnitude of both the direct and indirect effects is greater in the model where stress serves as a predictor of sleep problems, rather than the other way around. This suggests that stress has a greater impact on sleep problems than sleep problems have on stress.

## 4. Discussion

This study was conducted to clarify the relationship between stress and sleep quality. It examines whether the assumptions of the Cognitive Model of Insomnia [38] and the Hyperarousal Model of Insomnia [39], which propose that rumination and worry can mediate the relationship between stress and sleep quality, are valid in a healthy, non-clinical population of students. A panel design was employed to provide more complex conclusions about the relationships between these variables. We expected that individuals with a baseline tendency to ruminate or worry in response to perceived stressors would experience a greater disruption in sleep quality, and that poor sleep quality may lead to more stressful experiences through the same mechanism.

The results confirm our initial hypothesis about the bidirectional relationship between stress and sleep quality, and the direct path between stress and sleep. Stress has a greater impact on sleep problems than sleep problems have on stress, and stress and sleep problems often influence each other. Stress makes it difficult to fall asleep, and the inability to fall asleep increases the stress response [70]. Sleep deprivation and sleep disorders are associated with increased activity of the sympathetic nervous system and hypothalamic–pituitary–adrenal (HPA) axis activation, and so sleep disruption can lead to increased stress responsivity [71,72]. The bidirectional relationship between stress and sleep quality can be influenced by cortisol [70,73,74]. Research has demonstrated that shorter sleep duration and increased physical activity on the previous day lead to an increase in the cortisol awakening response (CAR), which refers to a biological increase in cortisol levels during the first 30 to 60 min after waking [73]. Disruptions in the natural secretion patterns of cortisol may affect sleep and overall health. This disruption affects the HPA axis, potentially leading to metabolic issues, heart diseases, and cognitive impairments [75]. These findings may explain the bidirectional relationship between disrupted sleep and stress. The asymmetry in this bidirectional relationship, where stress exerts a greater influence on sleep than vice versa, may be affected by external factors. Although poor sleep can heighten sensitivity to stress [71,72], the effect of sleep on stress may be less pronounced. This discrepancy may arise because stress among university students often originates from relationships with peers, teachers, and parents, as well as academic pressures [6], which are not directly related to sleep itself. In addition to the bidirectional relationship between sleep quality and stress, this study aimed to investigate whether rumination and worry, as forms of cognitive arousal, mediate this relationship. Insomnia is characterized by physiological, cognitive, or psychological arousal; however, the degree of correlation between sleep reactivity and hyperarousal is still unclear [43]. There is some evidence (e.g., [55]) that high levels of arousal can mediate the relationship between stress and sleep, with cognitive arousal accounting for more of the variance in this relationship than somatic arousal. This study finds that the relationship between stress and sleep quality is partially mediated by rumination. Cognitive arousal may result from high levels of rumination and worry tendencies [55]. According to Riemann’s model [39], arousal is expressed as somatic, cognitive, and cortical activity due to classical conditioning, abnormal processing of sensory stimuli and information, and long-term memory. Increased focus on sensory stimuli before sleep makes people with insomnia vulnerable to environmental stimuli, which hinders the initiation and maintenance of sleep. Information from memory related to environmental stimuli, the presence of arousal, and disturbed sleep can interfere with current experience and lead to negative expectations related to sleep, which in turn causes disrupted sleep [39].

Poor sleep quality in this study was associated with a greater tendency to ruminate, and increased rumination correlated with higher levels of stress. Additionally, elevated stress levels were associated with more rumination, which in turn predicted greater sleep disturbances. On the other hand, worry did not prove to be a mediator of the stress–sleep relationship. Worry proved to be a significant mediator only in parallel mediation, where the sleep quality–rumination–worry–stress path is significant, but only in the direction when disturbed sleep is a predictor of stress. The finding that rumination partially mediates the stress–sleep relationship was expected according to the theories set out in the Introduction [38,39] and the findings of other researchers [55,76]. According to Harvey’s Cognitive Model of Insomnia [38], individuals with insomnia often engage in excessive worry about their sleep and the potential consequences that lack of sleep will have on their health and daily functioning. This negative cognitive activity in the form of worry and rumination triggers autonomic arousal and emotional distress. Arousal is caused by the activation of the sympathetic nervous system, which prepares the body for stressful situations and induces a state of anxiety. This heightened state of anxiety and beliefs causes individuals to feel as though they have slept much less than they actually have. Consequently, they may believe that their daily functioning is much more impaired than it truly is. In other words, cognitive processes create a cycle in which a person becomes more and more worried and anxious about sleep issues, potentially culminating in excessive anxiety, physiological arousal, and real sleep deficits. According to the Cognitive Model of Insomnia, sleep problems are the result of these cognitive processes rather than actual deficits in the sleep–wake cycle [38]. Rumination among students can also be triggered by communication style. There is evidence that interpersonal peer relationships characterized by passive and catastrophic problem talk may become internalized as maladaptive, repetitive patterns of thinking [77]. These co-ruminations also negatively impact mental health [78] and may serve as an additional source of rumination among students.

Harvey’s [38] theory explains the mediating role of rumination in the relationship between stress and sleep quality, as anticipated. On the other hand, this theory and previous studies (e.g., [55]) also posited that worry would serve as a significant mediator in the relationship between stress and sleep quality; however, the results of this study did not support this expectation. It is possible that the reason for such a finding is the selection of the measure of worry. Some studies (e.g., [79]) show different effects of worry on sleep depending on the content of worry, whether it is a general tendency to worry or worrying about sleep-related content. The PSWQ used in this study is a general measure of worry, with no content exclusively related to sleep. It was found that general worry was not associated with impairments in sleep behavior; however, it did predict the disturbance and distress associated with insomnia. Conversely, sleep-related thoughts and concerns contribute to the severity and persistence of sleep difficulties [79]. Therefore, different worry measures that focus on sleep-related content could show different results. Students often worry about their academic obligations [6,57,58] and are prone to academic burnout [58]. While these obligations can be a significant source of stress, they are, in some respects, more manageable for students compared to other life events that are beyond their control. It is possible that academic worry has a smaller impact on disturbed sleep quality, which is why worry was not found to be a significant mediator in the sleep–stress relationship.

The effect of worry is significant only in parallel mediation; poor sleep quality leads to increased rumination, which in turn predicts more worry, and greater worry predicts more stress symptoms. Worry and rumination are distinct constructs, but both involve negative, repetitive thinking and share some similarities [80]. Rumination is characterized by prolonged, repetitive thinking about experiences, feelings, and also worries [49]. This raises the possibility that some differences between rumination and worry are artifacts of operationalization rather than distinctions between their natures [80]. The correlation between these constructs varies depending on the measures used. Measures of rumination that assess the thinking process in general, rather than in the context of depressive mood, are strongly correlated with worry [80]. This may provide the background for the relationship observed in our study, which utilized a measure of rumination that is not related to specific content.

The significance of this research is attributed to its panel design and the inclusion of college students, who are often a neglected population in studies on this topic. The study confirms that certain aspects of the Cognitive Model of Insomnia [38] and the Hyperarousal Model of Insomnia [39], which include rumination as a key process in the development of insomnia, are relevant for explaining the relationship between stress and sleep quality in a population without a diagnosis of insomnia. The results contribute to the existing literature by confirming the bidirectional relationship between sleep quality and stress symptoms, in addition to recognizing rumination as their underlying factor. These findings are important for the planning of preventive activities and therapeutic interventions. It was found that even brief online interventions aimed at reducing rumination and worry result in a decrease not only in repetitive negative thinking rumination but also in anxiety, depression, and distress [81].

Given the subjective nature of self-reported measures, it is important to consider the potential for recall bias. With regard to the subjectivity of self-reported measures and the differences in correlation between rumination and worry due to the use of different techniques, future studies should incorporate additional measures of rumination and worry, as well as more objective indicators of stress, such as cortisol levels. Another limitation of this study is that the effects of social jet lag on sleep quality were not investigated. Some research (e.g., [82]) has found that participants who experienced acute stress reported poor sleep quality. However, social jet lag (the discrepancy between sleep and wake times on weekdays compared to weekends) is also associated with acute stress. Exploring the relationship between sleep quality and stress by examining indicators such as weekend catch-up sleep and social jet lag would be essential. The concept of affective temperaments also appears to play a significant role in coping with stress [83], so this could also be included in future studies. The students involved in this study were not a clinical population, so they were mostly devoid of significant health or mental health problems. Participants with poorer physical and mental health, who may be at greater risk for stress and sleep-related problems, should be included in future research. Although the effect of gender was controlled in the analyses, the predominance of female participants may limit the generalizability of the findings. Future research should incorporate gender-balanced samples. The current study sample included undergraduate students from one university only, which limits the external validity and generalizability of our findings. For future studies, recruiting students from multiple universities is crucial and imperative. Additionally, this study was conducted at only two measurement intervals; the inclusion of at least three time points in future studies would likely result in more accurate and reliable outcomes. We suggest that future research employs longitudinal designs with at least three measurement points, controlling for biological variables such as cortisol and capturing potential effects of social jet lag on the relationship between stress and sleep.

## Figures and Tables

**Table 1:** Descriptive results and Pearson’s correlations between sleep quality (PSQI), stress symptoms (DASS-S), rumination (RS), and worry (PSWQ) measured in the first and second research waves.

	*M*_1_ ± *sd*_1_	*M*_2_ ± *sd*_2_	*t* (*df*)	1	2	3	4
1-PSQI	5.435 ± 2.759	5.527 ± 2.957	−0.580 (293)	0.644 **	0.389 **	0.274 **	0.228 **
2-DASS-S	15.209 ± 9.876	14.197 ± 9.327	1.574 (299)	0.450 **	0.617 **	0.424 **	0.599 **
3-RS	39.975 ± 6.975	38.818 ± 7.258	3.096 ** (290)	0.323 **	0.477 **	0.696 **	0.517 **
4-PSWQ	55.589 ± 14.490	54.182 ± 14.507	2.296 ** (287)	0.292 **	0.590 **	0.512 **	0.829 **

** *p* < 0.01, *M*_1_/*sd*_1_—first-wave data, *M*_2_/*sd*_2_—second-wave data, above diagonal—first-wave data correlations, under diagonal—second-wave data correlations, diagonal—retest correlation.

## Data Availability

The data presented in this study are available on request from the corresponding author. The data are not publicly available due to lack of funding.
